# Genome-wide analysis of *Streptococcus pneumoniae* serogroup 19 in the decade after the introduction of pneumococcal conjugate vaccines in Australia

**DOI:** 10.1038/s41598-018-35270-1

**Published:** 2018-11-16

**Authors:** Rebecca J. Rockett, Shahin Oftadeh, Nathan L. Bachmann, Verlaine J. Timms, Fanrong Kong, Gwendolyn L. Gilbert, Vitali Sintchenko

**Affiliations:** 1 Centre for Infectious Diseases and Microbiology - Public Health, Westmead Hospital, Westmead, 2145 Australia; 2 NSW Pneumococcal Reference Laboratory, Institute of Clinical Pathology and Medical Research – NSW Health Pathology, Westmead, 2145 Australia; 3 Centenary Institute, The University of Sydney, Camperdown, 2050 Australia; 4 Marie Bashir Institute for Infectious Diseases and Biosecurity, The University of Sydney, Camperdown, 2050 Australia

## Abstract

The decline in invasive pneumococcal disease (IPD), following the introduction of the 7-valent pneumococcal conjugate vaccination (PCV-7), was tempered by emergence of non-vaccine serotypes, particularly 19A. In Australia, three years after PCV-7 was replaced by PCV-13, containing 19A and 19F antigens, serogroup 19 was still a prominent cause of IPD in children under five. In this study we examined the evolution of serogroup 19 before and after introduction of paediatric vaccines in New South Wales (NSW), Australia. Genomes of 124 serogroup 19 IPD isolates collected before (2004) and after introduction of PCV-7 (2008) and PCV-13 (2014), from children under five in NSW, were analysed. Eleven core genome sequence clusters (cgSC) and 35 multilocus sequence types (ST) were identified. The majority (78/124) of the isolates belonged to four cgSCs: cgSC7 (ST199), cgSC11 (ST320), cgSC8 (ST63) and cgSC9 (ST2345). ST63 and ST2345 were exclusively serotype 19A and accounted for its predominantly intermediate penicillin resistance; these two clusters first appeared in 2008 and largely disappeared after introduction of PCV-13. Serogroup 19 was responsible for the highest proportion of vaccine failures in NSW. Relatively low immunogenicity of serogroup 19 antigens and Australia’s three-dose vaccine schedule could affect the population dynamics of this serogroup.

## Introduction

S*treptococcus pneumoniae* is a highly recombinant Gram positive bacterium which can be a commensal or pathogen in the respiratory tract^1^. Invasive pneumococcal disease (IPD) occurs when virulent strains enter the blood stream and cause a range of life-threatening syndromes, most importantly pneumonia, meningitis and sepsis. The burden of IPD is greatest in children under five years of age and the elderly, in whom it is associated with significant morbidity. Relatively few of the more than 90 serotypes of pneumococci are commonly associated with IPD and the polysaccharide capsule is considered the most important pneumococcal virulence factor^2^.

Antigens of the seven most common IPD-associated serotypes, including 19F, were included in the first polysaccharide-protein conjugate vaccine (PCV-7) that was publicly funded in 2005 for all Australian children under five years of age. The incidence of IPD declined dramatically, due to individual and herd immunity^3–5^, in countries where PCV-7 was widely used. In Australia IPD notifications in children under five fell from 55.4 to 16.8 cases per 100,000 population between 2004 and 2008^6,7^. However, this success was tempered by an increase in the absolute numbers and proportions of non-vaccine serotypes causing IPD, particularly 19A. In New South Wales (NSW), the most populous state of Australia, the number and proportion of cases due to 19A increased from nine of 245 (4%) in 2004 to 56 of 99 (57%) in 2008^6^. Similar increases in IPD incidence caused by serotype 19A were noted worldwide, often associated with high-level antibiotic resistance^8–10^. However, in Australia, most 19A IPD isolates were of intermediate penicillin resistance^11^.

The 13-valent polysaccharide-protein conjugate vaccine (PCV-13), which included serotype 19A antigen, replaced the PCV-7 vaccine in Australia in 2011. However, three years later in 2014, 27% (38/143: 29 serotype 19A; nine serotype 19F) of IPD cases in children under five in Australia were caused by serogroup 19, despite high vaccine coverage^12^. Pneumococcal conjugate vaccine failures are uncommon (2%), but those that occur are often due to serotype 19F^13^. This may be due to the relatively poor immunogenicity of the serotype 19F antigen^14^ and heterogeneity of the capsular (*cps*) biosynthesis locus in serogroup 19 which can reduce the spectrum of cross-protection provided by the serogroup 19 antigens contained in PCVs^15,16^.

Following introduction of PCV-7, ‘serotype switching’ or ‘capsular switch’ recombination was reported, particularly in serogroup 19^17^. These ‘capsular switch’ recombination events occurred when multi-drug resistant (MDR) clones recombined to switch their vaccine capsular (19F) to a non-vaccine, capsular type (19A)^16–19^. These recombination events were facilitated by genomic ‘hotspots’ on either side of the *cps* locus, which is flanked by the penicillin binding protein genes, *pbp*1a and *pbp*2x^20^, enabling pneumococci to evade both vaccine and antibiotic pressure. Recently, whole genome sequencing (WGS) has been used to examine this phenomenon seen as a potential threat to the long-term efficacy of pneumococcal vaccines^21^. WGS provides higher resolution strain typing and can identify recombination and genomic variability more effectively than multilocus sequence typing (MLST) or multilocus variable number of tandem repeats analysis (MLVA).

In this study, we applied WGS and genome-wide analysis to examine temporal diversity of serotypes 19F and 19A IPD isolates from children under five in NSW, Australia, in the year before (2004) introduction of PCV-7 and three years after the introduction of both PCV-7 and PCV-13 vaccines (2008 and 2014, respectively). In order to contextualize our analysis we also compared the genome sequences of Australian isolates with those of 152 serogroup 19 isolates from post-vaccine carriage studies in the UK and US^16,22^.

## Results

### Temporal changes in pneumococcal serotypes

Clinical data were available for 118 of 124 (94%) serogroup 19 IPD cases reported in NSW. Although PCV-7 was not publicly funded for all Australian children until January 2005, it was available for high-risk and Aboriginal and Torres Strait Islander children (a very small proportion of the population) from mid-2001. The number and distribution of serogroup 19 IPD isolates from children under five years of age in NSW between 2004 and 2016 are shown in Table 1. Antibiotic susceptibilities of isolates collected during the study period are summarised in Table 2 and related clinical data in Table 3.

**Table 1:** Serogroup 19 isolates from children <5 years of age, in NSW.

Serogroup 19/total (%)													
Year	2004	2005	2006	2007	2008	2009	2010	2011	2012	2013	2014	2015	2016
Total IPD isolates from children <5 yrs (all serotypes)	245^a^	141	71	81	99^a,b^	73	95	73	66	60	80^b^	58	63
Total serogroup 19	41^a^ (17)	33 (23)	21 (30)	35 (43)	60^a,b^ (61)	42 (58)	40 (42)	40 (55)	22 (33)	13 (22)	23^a,b^ (29)	19 (33)	10 (16)
Serotype 19F	32^c^ (13)	19 (14)	8 (11)	4 (5)	4^3,4^ (4)	5 (6)	1 (1)	3 (4)	2 (3)	—	7^4^ (9)	10 (17)	6 (10)
Serotype 19A	9^3^ (4)	14 (10)	13 (18)	31 (38)	56^c^ (57)	37 (51)	39 (41)	37 (55)	20 (30)	13 (22)	16^d^ (20)	9 (16)	4 (6)
Vaccine Failure^e^ - Serotype 19F	—	N/A	N/A	N/A	3 (3)	3 (4)	1 (1)	—	2 (3)	—	3^f,g^ (4)	7 (12)	4 (6)
Vaccine Failure^e^ - Serotype 19A	—	N/A	N/A	N/A	—	—	—	—	—	5 (8)	9^g,i^ (11)	6 (10)	4 (6)

Notes:

^a^Increase in number and proportion of serogroup 19 between 2004 and 2008; p = 0.0001.

^b^Decrease in number and proportion of serogroup 19 between 2008 and 2014: p < 0.01.

^c^Shift of predominant serotype from 19F to 19A after the introduction of PCV-7: p = 0.0001.

^d^Increase in number and proportion of serotype 19F after introduction of PCV-13 p < 0.01.

^e^Vaccine failure defined as IPD in children who had received the recommended 3 doses of PCV-7 and/or PCV-13.

^f^Two vaccine failures were in children with co-morbidities associated with increased risk of IPD.

^g^One vaccine failure case was reported in a child with co-morbidities associated with an increased risk of IPD.

^h^Two serotype 19F vaccine failure cases where fully vaccinated with PCV-7, one case was fully vaccinated with PCV-13.

^i^All 9 cases of 19A vaccine failure where fully vaccinated with PCV-13.

**Table 2:** Phenotypic antibiotic susceptibilities of serogroup 19 isolates from children <5 years of age, in NSW.

	Serogroup 19 (%)[19A]		
Year	2004	2008	2014
Total serogroup 19 isolates	41 [9]	60 [56]	23 [16]
MDR isolates	3 (7) [0]	5 (8)^a^ [3]	4 (17.4)^a^ [3]
Penicillin resistant (MIC <2 µg/mL)	5 (12) [0]	—	1 (4.3) [1]
Penicillin intermediate (MIC 0.12–1 µg/mL)	8 (20) [5]^b^	36 (60) [35]^b,c^	1 (4.3)^c^ [1]
Penicillin susceptible (MIC <0. 06 µg/mL)	25 (61) [3]	19 (32) [17]	17 (74) [11]

Notes:

^a^Small numbers of MDR isolates, but increase in proportion in 2014, mainly serotype 19A; difference not significant due to small numbers.

^b^Increase in penicillin intermediate isolates between 2004 and 2008, p = 0.0001.

^c^Decrease in penicillin intermediate isolates between 2008 and 2014, p = 0.0001.

MDR – multidrug resistant - resistant to penicillin, ceftriaxone and erythromycin: MIC – minimum inhibitory concentration.

**Table 3:** Clinical and vaccination data from IPD patients, <5 years of age in NSW, from whom IPD serogroup 19 isolates were isolated.

Demographic & core clinical data		2004	2008	2014
Total cases		n = 41	n = 60	n = 23
Median age, years (range)		1.45 (0.04–4.94)^a^	1.76 (0.0–4.73)	1.9 (0.0–5)
Specimen type	Blood^b^	40 (98%)	59 (98%)	21 (91.3%)
	Joint fluid	1(2%)	1 (2%)	—
	CSF	—	—	2 (8.7%)
Clinical presentation	Bacteremia	22 (54%)	31 (51.5%)	11 (47.8)
	Pneumonia	11 (27%)	25 (41.5%)	9 (39.1)
	Septic arthritis	1 (2%)	1 (2%)	—
	Meningitis	—	—	2 (8.7)
	Unknown	7 (17%)	3 (5%)	1 (4.3)
Vaccination History	Full	1 (2%)	50 (82%)	15 (65%)
	Partial	2 (5%)	6 (10%)	5 (22%)
	None	31 (76%)	1 (2%)	2 (9%)
	Unknown	7 (17%)	2 (3%)	1 (4%)
PCV-7 history	3 doses	1 (2.4%)	50 (83%)	5 (23%)
	2 doses	—	6 (10%)	1 (4%)
	1 dose	1 (2.4%)	1 (2%)	—
	No PCV-7	29 (70.8%)	2 (3%)	16 (70%)
	Unknown	9 (22%)	1 (2%)	1 (4%)
PCV-13 history	3 doses	—	—	10 (44%)
	2 doses	—	—	4 (17%)
	1 dose	—	—	1 (4%)
	No PCV13	—	—	7 (30%)
	Unknown			1 (4%)
PPV-23 history	1 dose	1 (2.4%)	—	—
Immune status	Compromised	3 (7%)	1 (2%)	2 (9%)
	Competent	32 (78%)	59 (98%)	20 (87%)
	Unknown	6 (15%)	—	1 (4%)
Indigenous status^c^	Non-indigenous	98%	95%	21 (91%)
	Indigenous	2%	5%	2 (9%)

Notes:

^a^Age unknown for 3 IPD cases in 2004.

^b^Clinical presentation of bacteremia includes an unknown number of cases of meningitis (lumbar puncture often performed after antibiotic therapy commenced).

^c^Aggregated percentage of IPD cases in Indigenous and non-indigenous populations where included in study years 2004 and 2008. In 2014, notified cases of IPD in Indigenous.

^d^Abbreviations: PCV-7; −13-7 or 13-valent pneumococcal conjugate vaccine; PPV-23-23-valent pneumococcal polysaccharide vaccine. and non-indigenous children is reported.

The total number of IPD cases in NSW, including those due to serogroup 19, fell significantly after introduction of each vaccine. However, the proportion of serogroup 19 isolates increased after the introduction of PCV-7 with an important shift from 19F to 19A as the predominant serotype (Table 1), and an associated significant increase in the proportion of penicillin intermediate isolates (Table 2). There were no significant increases in the incidence of human influenza cases during the years of the study (Supplemental Fig. S1). The subsequent introduction of PCV-13 led to an overall decrease in the proportion of serogroup 19 isolates, despite a small, but significant, increase in the proportion of 19F isolates (p < 0.01). In 2014, serogroup 19 isolates accounted for 29% (23/80) of isolates and, of these, 39% (9/23) were from cases of vaccine failure, the high prevalence of vaccine failures due to serogroup 19 continued and increased in 2015 (68% (13/19)) and 2016 (80% (8/10)) (Table 1). There were no significant differences in the age, clinical syndromes or immunisation histories of children with serogroup 19 IPD diagnosed in the three time periods of the study (Table 3).

### Core genome sequence clusters (cgSCs)

**Figure 1:**
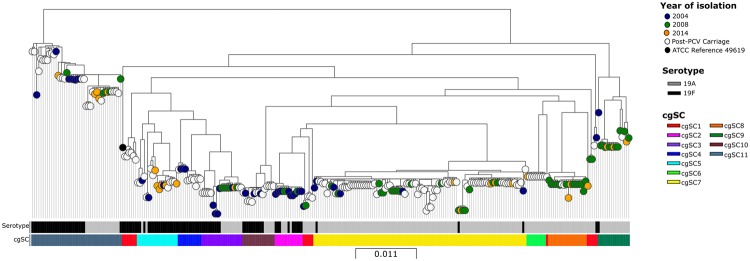
Maximum likelihood phylogeny of the 1330-gene core genome (cg) nucleotide alignment from 277 assembled genomes (124 from NSW, Australia, 105 from USA, 47 from UK and ATCC reference strain). Colour of isolate nodes denotes year collected and source (blue 2004; green 2008; orange 2014; white post-PCV carriage isolates from UK and USA and black ATCC *Streptococcus pneumoniae* strain 49619). Maximum likelihood phylogeny is separated into eleven sequence clusters (cgSCs) indicated by the colour of the first metadata block column. Serotypes of the isolates are denoted by the lower metadata block (black 19F; grey 19A).

**Figure 2:**
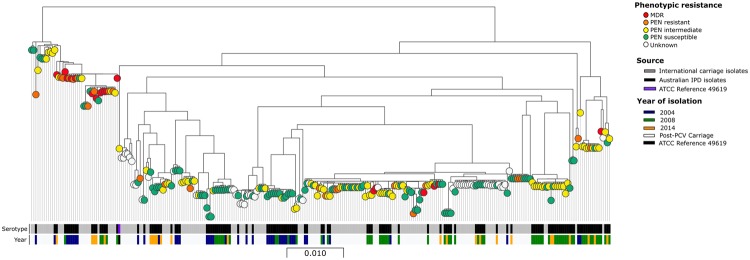
Phenotypic susceptibility of 277 serogroup 19 IPD isolates illustrated on the core genome phylogeny. Red nodes – multidrug resistant (MDR) isolates; i.e. phenotypically resistant to (MIC >2 µg/mL) to penicillin, ceftriaxone and erythromycin. Orange nodes – penicillin resistant isolates (MIC >2 µg/mL). Yellow nodes - penicillin intermediate resistant isolates (MIC 0.12–2 µg/mL). Green nodes - penicillin susceptible isolates (MIC <0.06 µg/mL g/L). White nodes - genomes from the international studies without phenotypic susceptibility data. The first column of metadata blocks depicts source of the isolate (black IPD isolates; grey international carriage isolates and purple ATCC *Streptococcus pneumoniae* strain 49619). The second column depicts year of isolation (blue 2004; green 2008; orange 2014; white post-PCV international carriage isolates and black ATCC *Streptococcus pneumoniae* strain 49619).

**Figure 3:**
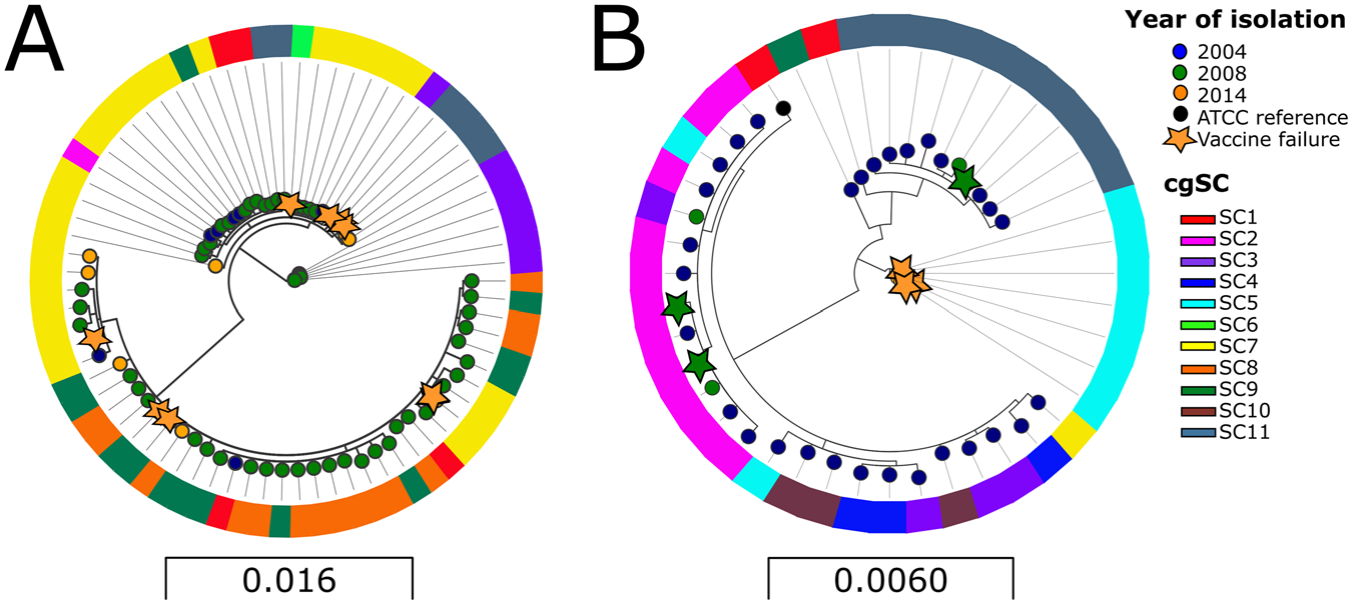
Maximum likelihood phylogenetic analysis of protein sequences from the 15 genes that form the *cps* locus from isolates collected in 2004 (blue nodes), 2008 (green nodes), and 2014 (orange nodes). Figure 3A demonstrates the amino acid sequence diversity of serotype 19A isolates and serotype 19F are depicted in Fig. 3B. Outer rings show core genome sequence clusters (cgSC) of isolates (see Fig. 1A). Isolates with the same *cps* phylogeny harbour highly diverse cgSC profiles. For both serogroups homology is noted between isolates collected in all years of the study. Stars indicate cases of vaccine failure, where subjects had received 3 doses of PCV-7 and/or PCV-13 (19F) and PCV-13 (19A).

Nucleotide alignments of 1330 core genes identified from 277 assembled genomes (124 from NSW, 105 from USA, 47 from UK and ATCC reference strain 49619) were used to construct a maximum likelihood phylogeny (Fig. 1). Eleven cgSCs were identified, one polyphyletic cluster (cgSC1) and 10 monophyletic clusters (cgSCs 2–11). The distribution of cgSCs and their relationship to multilocus sequence types (STs) are shown in Table 4. The majority of monophyletic clusters contained both 19F and 19A isolates from all three years of the study; exceptions were cgSCs 8 and 9, which first appeared in 2008 and contained only 19A and cgSC7, which predominantly contained serotype 19A in post-vaccine isolates. There were 15 documented vaccine failure cases of IPD in fully vaccinated children (six attributed to 19F; nine to 19A), i.e. they had received three doses of either PCV-7 or PCV-13 (19F isolates) or PCV-13 (19A isolates) (Table 1). The 15 isolates were distributed among cgSCs 2, 3, 5, 7, 9 and 11 (Table 4 and Fig. 3). Phenotypic antibiotic susceptibilities where displayed on the core genome phylogeny, Australian MDR isolates were only contained in cgSC11 (Fig. 2).

**Table 4:** Core genome sequence clusters (cgSCs), multi-locus sequence types (STs) and serotypes of NSW IPD isolates in pre- and post-vaccine years.

cgSC (cases)	ST (cases)	Serotypes 2004		Serotypes 2008		Serotypes 2014	
		19F (n = 32)	19A (n = 8)	19F (n = 4)	19A (n = 56)	19F (n = 7)	19A (n = 16)
cgSC1 (6)^1^	81 (2)		1		1		
	2417 (2)				2		
	242 (1)	1					
cgSC2 (12)	66 (13)	8	2	2 (VF n = 2)	1		
cgSC3 (12)	1201 (9)	1	1		6		1 (VF)
	9 (1)	1					
	New^a^ (2)	2					
cgSC4 (3)	309 (3)	3					
cgSC5 (9)	654 (5)	1				4 (VF n = 3)	
	3616 (1)					1	
	251 (1)					1	
	476 (1)		1				
	482 (1)	1					
cgSC6 (1)	695 (1)						1(VF)
cgSC7 (31)	199 (15)	2	2		9		2 (VF n = 1)
	1756 (2)		1		1		
	876 (5)		1		4		
	416 (4)				3		1 (VF)
	649 (2)				2		
	667(1)						1
	419(1)						1
	3017(1)				1		
cgSC8 (16)	63(17)				15		2
cgSC9 (14)	2345 (7)				5		2 (VF n = 2)
	277 (1)				1		
	172 (1)				1		
	339 (1)	1					
	New^b^ (2)				1		1
cgSC10 (3)	162 (3)	3					
cgSC11 (18)	320 (13)	6		1	3		3 (VF n = 2)
	271 (1)					1	
	236 (1)	1					
	352 (1)	1					
	1451 (1)						1 (VF)
	Undetermined^c^ (1)			1(VF n = 1)			

Notes:

^a^Contains ATCC *Streptococcus pneumoniae* strain 49619, isolated in 1992.

^b^New - allelic profile not previously recognized or designated as a ST (2 isolates in cgSC3, and 2 isolates in cgSC9).

^c^Undetermined - STs could not be generated for one isolate because of housekeeping gene deletion in *ddl* (1 isolate, cgSC11).

### Multilocus sequence types (STs)

The 124 IPD isolates from NSW included 35 STs (two novel STs and one isolate with a *ddl* deletion), as shown in Table 4 with the corresponding cgSCs. Five cgSCs included single STs (cgSCs 2, 4, 6, 8 and 10). However the MLST did not accurately predict the homology of other cgSCs. No MLST alleles were shared between STs in polyphyletic cgSC1. The majority of isolates in cgSC11 were ST320 but other STs in this cluster did not share a common MLST allele. cgSCs 3, 5 and 7 shared a single common allele and STs in cgSC9 were triple locus variants (TLV). cgSC7 was the most diverse, containing eight distinct STs spread over all target years, which were predominantly serotype 19A in 2008.

The number of STs among serotype 19F isolates decreased along with overall numbers, following the introduction of PCV-7 (12 STs in 2004 versus two in 2008) while the number of 19A isolates and STs increased (seven STs in 2004 versus 13 in 2008). The number of STs detected in 2014 decreased again for serotype 19A isolates, from 13 in 2008 to 10 in 2014; STs detected for serotype 19F isolates increased from two in 2008 to four in 2014.

### Amino acid variation within the cps loci

Maximum likelihood phylogeny of the 15 proteins encoded by the *cps* locus genes showed considerable heterogeneity between isolates of both serotypes, and this heterogeneity did not change between target years (Fig. 3). In serotype 19F, the greatest variation was in amino acid sequences of glucose phosphate transferase (WchA or CpsE). Low homology among amino acid sequences of dTDP-4-dehydrorhamnose reductase (RmlD) between serotype 19A isolates probably resulted from a recombination event that caused an inversion of *rml*D. Amino acid sequences of the 15 vaccine failure isolates (six 19F; nine 19A) did not cluster within a particular phylogenetic clade or share any conserved non-synonymous changes within either serotype. Isolates with the same *cps* phylogeny also harboured highly diverse cgSC profiles. These findings indicate that vaccine failures are unlikely to be caused by a particular change in the vaccine antigens or an individual cgSC.

## Discussion

In this study we have shown that the evolution of serogroup 19, after the introduction of PCV vaccines was different in Australia, from that described in some other countries^5,17,23^ and was associated with less antibiotic resistance^11^. Our genomics data suggests that serotype 19 antigen heterogeneity may contribute to vaccine failure, due to serogroup 19 but the high rate, in Australia cannot be explained by a single serotype 19A or 19F antigen variant or common cgSC.

Although widespread uptake of PCVs have significantly reduced the incidence of IPD in children under five years of age in Australia and elsewhere, serogroup 19 remained the predominant cause of IPD in NSW (13 of 62 [21%] cases in 2015–16; Table 1), despite the fact that 93.5% of Australian children were fully vaccinated with PCV-7 and/or PCV-13, by 5 years of age in 2015^24^. The success of PCV-7 vaccine was partially offset by the rapid emergence of serotype 19A; its incidence fell and that of serotype 19F increased, after the introduction of PCV-13. Our findings indicate that the increased prevalence of intermediate penicillin resistance in serotype 19A^11^, post PCV-7, was largely due to emergence of cgSC8/ST63 and cgSC9/ST2345 and expansion of cgSC7/ST199, nearly all of which had intermediate penicillin resistance. Although cgSC8 and 9 were prominent among NSW isolates, particularly in 2008, they were rarely detected, if at all, among published isolates, from USA and UK, with which we compared them (cgSC8 - USA 2/106; UK 0/48; cgSC9 - USA 0/106; UK0/48). Rather, serotype 19A, cgSCs 7 and 11 were the predominant post-PCV-7 clones in the UK and USA, including high-level penicillin resistant clones cgSC7 (ST695) and MDR clone cgSC11 (ST320). There was only a single ST695 (cgSC6) isolate among our study isolates and, unlike those from the USA, it was not penicillin resistant. We note that USA and UK isolates in that comparison were colonising, rather than IPD strains. However, it is unlikely that this difference would affect strain distribution significantly and our findings are consistent with reported studies of post-PCV IPD serotype distribution in UK and USA^10,25,26^. Both cgSC8 and 9 decreased substantially after the introduction of PCV-13, associated with a fall in overall intermediate penicillin resistance.

Following the removal of highly successful vaccine serotype clones, in the northern hemisphere, the prevalence of a previously unrecognised clone MDR 19A/ST320 (cgSC11 in our study)^8,25–27^ increased. This was apparently due to a capsular switch from 19F to 19A, which probably occurred independently of vaccine use^15,16,28^. In contrast, although MDR 19F/cgSC11/ST320 was present in NSW before the introduction of PCV-7, as shown in this study, its prevalence did not increase afterwards. The prominent 2008 serotype 19A clones in NSW, cgSC8/ST63 and cgSC9/ST2345 (Fig. 1) have intermediate, rather than high level, penicillin resistance. We identified small numbers of ‘capsular switch’ 19A/ST320 variants and closely related STs in cgSC11, in both 2008 and 2014 (Table 4). The phylogeny of cgSC11 illuminated the post-PCV emergence of serotype 19A pneumococci: serotype 19F isolates are contained in ancestral branches and serotype 19A in more divergent branches, which is consistent with all pre-PCV-7 cgSC11 isolates expressing serotype 19F, whereas most post-PCV cgSC11 isolates were 19A. Recent population studies of *S. pneumoniae* suggest that the rate at which recombination occurs can differ between strains; the highest recombination rate among all encapsulated pneumococci is in ST320/cgSC11, in which transformation events are predicted to occur every one to two years^16^.

Antibiotic pressure can be a strong driver of recombination in pneumococci whereby *pbp* genes in penicillin susceptible strains are replaced by *pbp* genes carrying mutations that confer penicillin resistance, in the presence of high antibiotic consumption^20^. Several other countries, including Germany and Norway, which both have relatively low antibiotic usage^29^, have also reported a relatively low MDR ST320 prevalence, raising the possibility that its prevalence could be related to national antibiotic usage^9,30^. However, this is an unlikely explanation of the low prevalence in Australia, where the antibiotic prescribing rates in the community are higher than in many European countries^29^, including France, which reported expansion of the MDR 19A/ST320 clone^31,32^.

Most cases of IPD due to serotype 19F in 2008 and 2014, were vaccine failures including three of the four in 2008 and three of seven cases in 2014. A recent systematic review suggested that vaccine failures following PCV-7, were rare, overall (2.1%), but 38% of them were caused by serotype 19F and commonly occurring in children with underlying co-morbidities (42.9%)^13^. The vaccine failure rate due to 19F in our cohorts of children under 5 years was comparable to that in other studies (3% in 2008; 4% in 2014). Fewer international studies have reported vaccine failure rates after introduction of PCV-13. In our study, 11% (nine of 80) of IPD cases in 2014, were vaccine failures due to serotype 19A. In Australia, overall, 23% (32 of 141) of IPD cases, in 2014, were vaccines failures and, of these, half (16; 11% of total cases) were due to serotype 19A; the other main causes were serotypes 3 (10; 7%) and 19F (four; 3%)^12^. Vaccine failure rates in Australia exceeded post-PCV rates reported in France and Spain (<3%)^33,34^. Unlike other PCV vaccine failure studies, our data indicated that only three of 15 vaccine failures were in children with underlying co-morbidities.

The high vaccine failure rate due to serogroup 19 could be associated with heterogeneity of the serogroup 19 *cps* locus^35^ which may mean that immune responses to 19F and 19A strains used in PCV vaccines do not protect against all serogroup 19 stains. Diversity among *cps* loci found in this study was largely due to recombination within the 19A *rml* operon and 19F *wch*A gene, both of which have been demonstrated in serogroup 19 previously and are not thought to increase virulence or fitness^28,36^. Such within-serogroup recombinations have been recently reported to be more common than ‘capsular switching’ where the entire *cps* locus is replaced^28^. However, isolates from vaccine failures, in this study, did not share common amino acid sequence changes in serotype antigens or a common cgSC. This suggests that, while pathogen heterogeneity may contribute to vaccine failure, post-PCV evolution cannot fully not explain the increased incidence of vaccine failures due to serogroup 19. On the other hand, higher anticapsular antibody titres are required for efficient opsonophagocytosis of serotype 19F strains^37,38^. This could explain the higher vaccine failure rate associated with Australia’s three-dose vaccine schedule compared with those in countries with the more usual four-dose schedule^39^.

In conclusion, this is the first comparison of IPD in Australia before and after introduction of PCV using next generation sequencing. Although it was limited to a single Australian state (NSW has approximately one third of Australia’s population) and selected years, our findings have shown significant evolution of serogroup 19 in response to introduction of pneumococcal conjugate vaccines, which differs from that reported in some other countries. Substantial replacement of serotype 19F with serotype 19A occurred, following introduction of PCV-7, due to expansion of predominantly penicillin intermediate clones (mainly cgSCs 7, 8 and 9), and largely disappeared after the introduction of PCV13. By contrast, in the USA and UK, emergence of serotype 19A was associated with high-level penicillin resistance (mainly cgSCs 7 and 11). We have documented higher rates of vaccine failure due to serogroup 19 than previously described elsewhere. While this may be partly attributed to a high level of diversity among serogroup 19 *cps* loci, we hypothesise that Australia’s three-dose PCV-13 vaccine schedule, rather than the three-dose plus booster schedule used in many other countries, may have played a major role.

## Materials and Methods

### Source of isolates

All serogroup 19 IPD isolates from children under 5 years of age, referred by public and private pathology providers to the NSW Pneumococcal Reference Laboratory (PRL) in Sydney for public health surveillance of pneumococcal disease including serotyping and molecular typing of isolates. Historical isolates collected in the years 2004, 2008 and 2014 were included in the study. The ATCC serotype 19F strain 49619 was sequenced as the reference. Two isolates were excluded from analysis in each of 2008 and 2014 sets (i.e. two isolates could not be located, and two isolates had an incorrect serotyping result).

### Clinical and demographic data

Data for each case, including age, sex, diagnosis, specimen type, vaccination history and comorbidities were retrieved from NSW Health Notifiable Conditions Information Management System (NCIMS), Communicable Diseases Branch, Health Protection NSW. Vaccine failure cases where defined as IPD occurring in children who had received three doses of PCV-7 or PCV-13 (for vaccine failures due to 19F) or PCV-13 (19A). The study was approved by the NSW Population & Health Services Research Ethics Committee (HREC/18/CIPHS/28). Descriptive statistics where conducted using a two tailed Fisher’s exact test to measure variance in IPD cases over study years.

### Serotyping and phenotypic antibiotic susceptibility testing

Serotyping was performed by Neufeld’s Quelling test, using pool, type and factor specific antisera (Statens Serum Institut, Copenhagen, Denmark). Minimum inhibitory concentrations (MICs) for penicillin, ceftriaxone and erythromycin where measured using broth microdilution and interpreted according to EUCAST criteria^11^. Multidrug resistant (MDR) isolates were defined as those that were phenotypically resistant to penicillin (MIC >2 mg/L), ceftriaxone and erythromycin.

### Nucleic acid extraction and library preparation

Isolates were thawed from −80 °C storage in STGG (skim milk-tryptone-glucose-glycerin) and cultured on horse blood agar overnight at 37 °C, 5% CO_2_. Subculture was performed to ensure purity. A McFarland number 3 suspension was prepared in nucleic acid free H_2_0 for DNA extraction. Extraction was performed in accordance with manufacturer’s instructions using the Blood and Tissue Mini Kit (Qiagen, Australia) for Gram positive bacteria as per the manufacturer’s instructions. DNA extracts were treated with 1 Unit of RNase. Total DNA concentration was quantified using Picogreen (Invitrogen, Australia) and 1 ng/µL of DNA was used to prepared DNA libraries in accordance with manufacturer’s instructions employing the Nextera XT Library Preparation Kit. Multiplexed libraries were sequenced using paired-end 150-bp chemistry on the NextSeq. 500 (Illumina, Australia). All methods were carried out in accordance with The University of Sydney’s institutional guidelines and regulations.

### International IPD genomes

Fastq files were downloaded from two international studies that investigated the changes in pneumococcal population structure after the introduction of PCV-7 and PCV-13 vaccination in the UK and USA^16,22^. A total of 152 serotype 19 carriage isolates were included in our study (47 and 105 isolates from the UK and the USA studies, respectively); raw reads were processed through the bioinformatics pipeline as described below (European Nucleotide Archive study accessions: PRJEB2417 and PRJEB2632).

### Bioinformatic analysis

De-multiplexed sequencing reads were trimmed, based on a minimum quality read score of 20, and trimmed^40^ reads were *de novo* assembled using SPAdes^41^. The quality of de novo assemblies was accessed with Quast^42^, only contigs over 1000-bp in length with a minimum coverage of 50 reads were included in further analysis. Final contigs were annotated with Prokka^43^. MLST was inferred from final contigs using the *S. pneumonia* MLST scheme (http://pubmlst.org/spneumoniae/). Core genome analysis was conducted using Roary: the pan genome pipeline^44^. Maximum-likelihood phylogeny of the pan genome was assessed with RAxML with 100 bootstrap replicates^45,46^. Core genome alignments were employed to cluster isolates using the hierarchical Bayesian Analysis of Population Structure (hierBAPS) software^47^. Phylogeny and metadata were visualised with Microreact and Inkscape^48^. Raw sequencing reads were deposited in the European Nucleotide Archive; project accession PRJEB28571(Supplemental Table 1).

### Genomic variation in the pneumococcal capsular polysaccharides

The amino acid sequence of 15 genes between the putative transcriptional regulator YwtF (also known as *wzg* or *cps*A) and the dTDP-4-dehydrohamnose reductase gene (*str*L or *rfb*D) were extracted from contigs using BLAST+ and defined as the capsular biosynthesis locus that encodes the pneumococcal capsular polysaccharide (*cps*)^49^. The sequence of *cp*s genes were concatenated and aligned using the multiple sequence alignment tool MAFFT. Maximum likelihood phylogeny was constructed by RAxML software with 100 bootstrap replicates^45,46^. Visualization and comparison of potential recombination sites was performed using BratNextGen^50^.

## Electronic supplementary material

Supplemental Table 1

Supplemental Figure 1.
