# Supplementary material for: Genome-wide analysis of Streptococcus pneumoniae serogroup 19 in the decade after the introduction of pneumococcal conjugate vaccines in Australia
Source: Sci Rep. 2018 Nov 16;8:16969. doi: 10.1038/s41598-018-35270-1 (PMC6240094; doi:10.1038/s41598-018-35270-1)
Supplement: Supplementary file 2 — Supplemental Figure 1. [file 41598_2018_35270_MOESM2_ESM.docx]

**Genome-wide analysis of *Streptococcus pneumoniae* serogroup 19 in the decade after the introduction of pneumococcal conjugate vaccines in Australia**

Rebecca J. Rockett, Shahin Oftadeh, Nathan L. Bachmann, Verlaine Timms, Fanrong Kong, Gwendolyn L. Gilbert, and Vitali Sintchenko

**Supplemental Figure 1. Notified cases of influenza and IPD in Australia in children under 5 years of age**

**
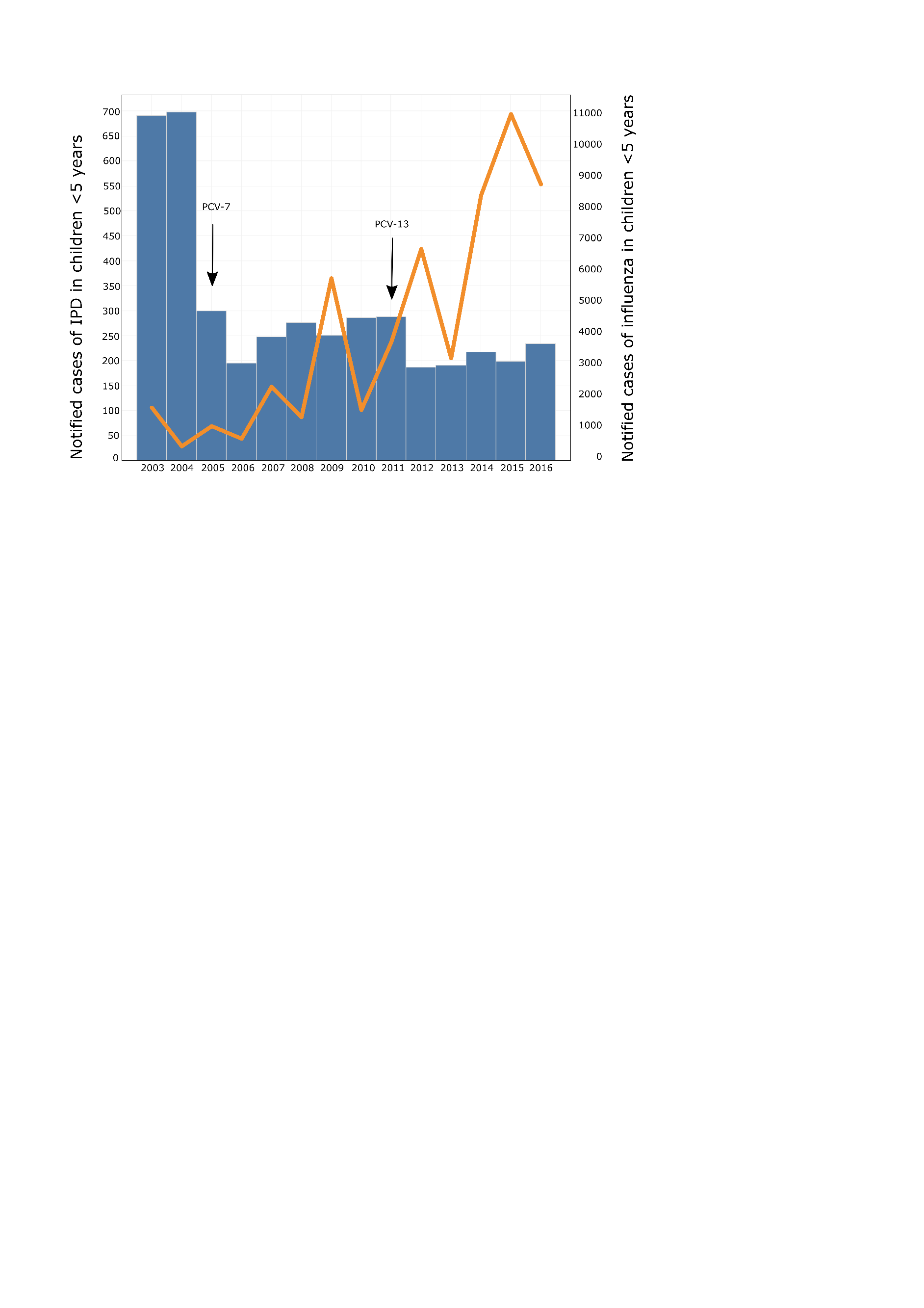
**

**Figure S1.** Notified cases of influenza (yellow line) and IPD (blue bars) in Australian children <5 years of age for the years 2003 to 2016. Black arrow indicates the time of introduction of pneumococcal conjugate vaccines into the routine childhood immunisation schedule.
